# Enhancing gestational diabetes mellitus risk assessment and treatment through GDMPredictor: a machine learning approach

**DOI:** 10.1007/s40618-024-02328-z

**Published:** 2024-03-09

**Authors:** J. Xing, K. Dong, X. Liu, J. Ma, E. Yuan, L. Zhang, Y. Fang

**Affiliations:** 1 Department of Laboratory Medicine, The Third Affiliated Hospital of Zhengzhou University, 7 Kangfu Qian Street, Zhengzhou, 450052 Henan People’s Republic of China; 2 Zhengzhou Key Laboratory for In Vitro Diagnosis of Hypertensive Disorders of Pregnancy, Zhengzhou, 450052 People’s Republic of China

**Keywords:** Gestational diabetes mellitus, Machine learning, Risk prediction, Treatment recommendation

## Abstract

**Background:**

Gestational diabetes mellitus (GDM) is a serious health concern that affects pregnant women worldwide and can lead to adverse pregnancy outcomes. Early detection of high-risk individuals and the implementation of appropriate treatment can enhance these outcomes.

**Methods:**

We conducted a study on a cohort of 3467 pregnant women during their pregnancy, with a total of 5649 clinical and biochemical records collected. We utilized this dataset as our training dataset to develop a web server called GDMPredictor. The GDMPredictor utilizes advanced machine learning techniques to predict the risk of GDM in pregnant women. We also personalize treatment recommendations based on essential biochemical indicators, such as A1MG, BMG, CysC, CO2, TBA, FPG, and CREA. Our assessment of GDMPredictor's effectiveness involved training it on the dataset of 3467 pregnant women and measuring its ability to predict GDM risk using an AUC and auPRC.

**Results:**

GDMPredictor demonstrated an impressive level of precision by achieving an AUC score of 0.967. To tailor our treatment recommendations, we use the GDM risk level to identify higher risk candidates who require more intensive care. The GDMPredictor can accept biochemical indicators for predicting the risk of GDM at any period from 1 to 24 weeks, providing healthcare professionals with an intuitive interface to identify high-risk patients and give optimal treatment recommendations.

**Conclusions:**

The GDMPredictor presents a valuable asset for clinical practice, with the potential to change the management of GDM in pregnant women. Its high accuracy and efficiency make it a reliable tool for doctors to improve patient outcomes. Early identification of high-risk individuals and tailored treatment can improve maternal and fetal health outcomes http://www.bioinfogenetics.info/GDM/.

**Supplementary Information:**

The online version contains supplementary material available at 10.1007/s40618-024-02328-z.

## Introduction

Gestational Diabetes Mellitus (GDM) is defined as glucose intolerance that is first diagnosed during pregnancy and can have serious consequences for both the mother and the baby [1]. GDM is characterized by impaired glucose tolerance that typically occurs during the second or third trimester of pregnancy. GDM is associated with various adverse maternal and fetal outcomes, including preeclampsia, preterm birth, macrosomia, and neonatal hypoglycemia [2]. Moreover, women with a history of GDM are at an increased risk of developing type 2 diabetes [3].

The diagnosis of GDM is typically based on the results of an oral glucose tolerance test (OGTT) performed between 24 and 28 weeks of gestation. The criteria for diagnosing GDM have evolved, with the most recent guidelines from the International Association of Diabetes and Pregnancy Study Groups (IADPSG) recommending lower glucose thresholds for diagnosis [4]. However, the cost-effectiveness of the IADPSG criteria has been questioned, and alternative diagnostic criteria have been proposed [5].

Accurate prediction of GDM risk is essential for early identification and intervention to prevent adverse outcomes. However, predicting GDM risk can be challenging due to the complex interplay of various risk factors, including maternal age, BMI, family history of diabetes, previous history of GDM, and ethnicity [6–9]. Moreover, the accuracy of GDM risk prediction is limited by the use of static risk factors, which do not account for dynamic changes in clinical parameters during pregnancy. Providing personalized treatment recommendations for GDM is also challenging due to the heterogeneity of the condition and the lack of consensus on optimal management strategies. Current management strategies for GDM include dietary modifications, physical activity recommendations, and medication options [10]. However, the optimal treatment approach for individual patients is often unclear, and there is a need for personalized treatment recommendations based on the patient's individual risk profile and clinical history.

Machine learning has become an increasingly popular tool for predicting and diagnosing medical conditions, including GDM. By analyzing large datasets of patient information, machine learning algorithms can identify patterns and risk factors that may not be immediately apparent to human clinicians. This can lead to earlier and more accurate diagnoses, as well as more personalized treatment plans. Predicting the risk of GDM can be divided into three categories based on the different features used. First, clinical variables-based models [11, 12] are based on maternal personal information, medical history, and history of the patient. Second, biochemical variables-based models [13, 14] are based on biomarkers that are measured in maternal biological fluids in the blood. Third, clinical and biochemical variables-based models [15, 16]. Studies have demonstrated the potential of machine learning for predicting GDM risk and improving patient outcomes. For example, Xiong et al. found that a support vector machine (SVM) algorithm had the highest accuracy in predicting GDM risk, with an area under the curve (AUC) of 0.84 [17]. Wang et al. (2020) found that a random forest algorithm had the highest accuracy in predicting GDM risk, with an AUC of 0.777 [18]. By incorporating machine learning algorithms into clinical practice, healthcare providers may be able to identify at-risk patients earlier and provide more personalized care.

The purpose of this study is to introduce the GDMPredictor web server, a novel tool for predicting GDM risk and providing personalized treatment recommendations based on the patient's individual risk profile and clinical history. The GDMPredictor web server uses a machine learning algorithm that integrates biochemical and clinical parameters to generate a comprehensive risk score for GDM. The GDMPredictor web server provides personalized treatment recommendations based on the patient's individual risk profile and clinical history, including appropriate dietary modifications, and physical activity recommendations. The web server is accessible from any device with an Internet connection and provides a patient-friendly interface that allows patients to input their data and receive personalized recommendations in real time.

## Materials and methods

### Data collection and preprocessing

In this study, a total of 3681 pregnant women were recruited from the Third Affiliated Hospital of Zhengzhou University in the year 2019. Among them, 1500 were diagnosed with gestational diabetes mellitus (GDM). The data collection was carried out by trained healthcare professionals who obtained informed consent from each participant. The following information was collected from each participant: (1) Clinical history: adverse pregnancy (AP), Intrahepatic cholestasis of pregnancy (ICP), Thyroid Diseases (TD), eclampsia, twins, days of pregnancy (Day), age, BMI (body mass index). (2) Biochemical: alanine aminotransferase (ALT), aspartate aminotransferase (AST), gamma-glutamyl transferase (GGT), alkaline phosphatase (ALP), total protein (TP), albumin (ALB), total bilirubin (TBIL), direct bilirubin (DBIL), total bile acid (TBA), blood urea nitrogen (UREA), creatinine (CREA), uric acid (UA), blood glucose (BMG), alpha-1 microglobulin (A1MG), Cystatin C (CysC), Carbon dioxide (CO2), and fasting plasma glucose (FPG). The data were preprocessed to ensure accuracy and consistency. First, missing values were checked and handled accordingly. Second, outliers were identified and removed using statistical methods. Third, the collected data were standardized to remove the influence of measurement units and to improve comparability between variables. The processed dataset for machine training is reported in Supplementary Table 1.

To ensure data quality, data entry and cleaning were performed by two independent researchers. In cases of discrepancies, a third researcher was consulted to resolve differences. All data were stored securely and analyzed using appropriate statistical software. Overall, the data collection and preprocessing procedures used in this study were designed to ensure high-quality and reliable data, which will facilitate the analysis and interpretation of the results.

### Feature selection and engineering

The feature selection was conducted to identify the most important predictors of GDM. Several feature selection methods were employed, including correlation analysis, univariate analysis, and recursive feature elimination. Clinical and biochemical features with high correlation to the outcome variable (GDM) were retained, while those with low correlation were removed. Recursive feature elimination was used to identify the subset of features that provided the best predictive performance. In addition, the Shapley values (SHAP) [19] and Uniform Manifold Approximation and Projection for Dimension Reduction (UMAP) [20] were utilized to explain the feature selected. Moreover, the selected features were used to train machine learning models. Overall, the feature selection and engineering methods used in this study were designed to identify the most informative predictors of GDM and to create new features that might improve the accuracy of the predictive model. These methods will facilitate the development of a robust and accurate machine learning model for GDM prediction. Finally, we selected 8 clinical features and 7 biochemical features as input for the GDMPredictor web server.

### Machine learning algorithms used for prediction

In this study, we employed various machine learning algorithms to predict the outcome variable based on the input features. A range of models was tested, including Gaussian NB, random forest, k-neighbors, AdaBoost, Gradient boosting, Bernoulli NB, Decision tree, and support vector machines. These methods were implemented by SciKit-learn package [21] with Python language. The models were trained using a combination of the selected features and hyperparameters tuning and evaluated using a variety of performance metrics, such as accuracy, precision, recall, and area under the curve (AUC). By comparing the results of different algorithms, we identified the most suitable method for our problem and developed a predictive model with high accuracy and robustness. The machine learning algorithms used in this study have proven to be effective in predicting the outcome variable based on the input features and can be applied in various fields, such as healthcare, finance, and marketing to solve real-world problems.

### Evaluation metrics

In this study, we aimed to predict GDM risk using a machine learning model with tenfold cross-validation and evaluate the model using various evaluation metrics. The model was trained on 9 of the 10 subsets and tested on the remaining subset. This process was repeated 10 times, with each subset being used as the test set once. The performance of the model was evaluated using the following evaluation metrics: the true-positive rate (TPR, recall) (1), precision (positive predictive value, PPV) (2), false-positive rate (FPR) (3), accuracy (ACC) (4), and F1 score (5) calculate as follows:

1 $${\text{Recall}}= {\text{TPR}}= \frac{{\text{TP}}}{{\text{TP}}+{\text{FN}}}$$

2 $${\text{precision}}={\text{PPV}}= \frac{{\text{TP}}}{{\text{TP}}+{\text{FP}}}$$

3 $${\text{FPR}}= \frac{{\text{FP}}}{{\text{FP}}+{\text{TN}}}$$

4 $${\text{ACC}}= \frac{{\text{TP}}+{\text{TN}}}{{\text{TP}}+{\text{TN}}+{\text{FP}}+{\text{FN}}}.$$

Using a tenfold cross-validation approach and evaluating the model using multiple evaluation metrics, we aimed to ensure that the model was robust and not overfitting to the data.

## Results

### Overview of the GDMPredictor

**Fig. 1:**
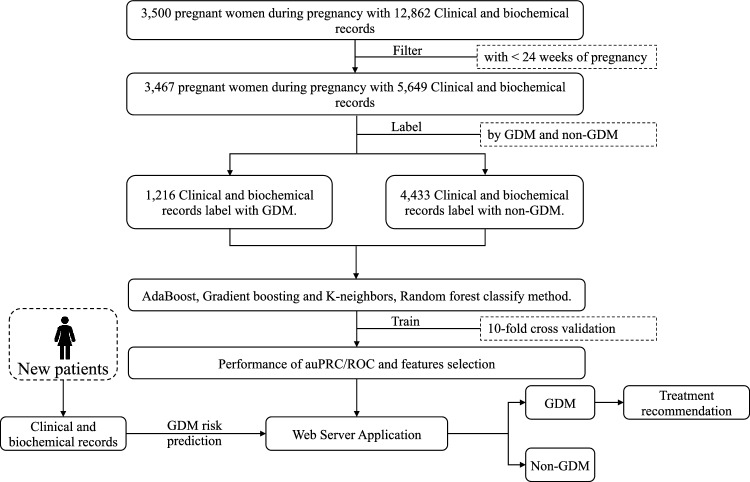
Schematic representation of the workflow of the GDMPredictor

The workflow of the GDMPredictor is shown in Fig. 1. GDMPredictor is an innovative predictive tool designed to assist healthcare professionals in predicting the risk of gestational diabetes mellitus (GDM) in pregnant women. The tool was developed based on a dataset that included more than 3500 pregnant women, whose clinical and biochemical records were filtered to include only those with less than 24 weeks of pregnancy. From this dataset, over 3400 pregnant women were selected for further analysis, and their clinical and biochemical records were labeled either as GDM or non-GDM. To train the tool, the dataset was divided into over 1200 records labeled as GDM and over 4400 records labeled as non-GDM. Eight classification methods were employed to train the GDMPredictor tool, namely Gaussian NB, random forest, k-neighbors, AdaBoost, Gradient boosting, Bernoulli NB, Decision tree, and support vector machines. The performance of each model was evaluated using tenfold cross-validation and AUC and the area under the precision–recall curve (auPRC) as metrics. The best model was selected based on these metrics, and feature selection was carried out using the SHAP value. The GDMPredictor tool was subsequently developed into a web server application that can be used to predict the risk of GDM in new patients. The tool provides valuable information that can assist healthcare professionals in managing the condition and improving maternal and fetal outcomes.

### Clinical and biochemical features

**Fig. 2:**
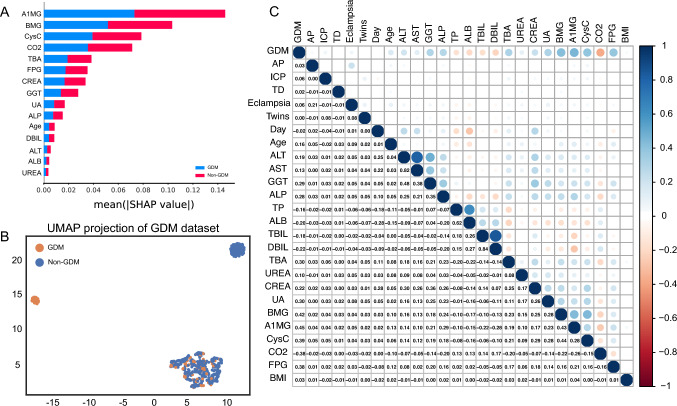
Clinical and biochemical features of the GDM dataset. **A** The SHAP value elucidates the significant features. **B** The UMAP demonstrates the collected GDM dataset. **C** The Pearson correlation coefficient shows the correlation between the clinical and biochemical features

In this study, we aimed to predict the risk of gestational diabetes mellitus (GDM) using 8 clinical information and 18 biochemical criteria as features. To detect the most important features for prediction, we utilized SHAP [19] which revealed that A1MG, BMG, CysC, CO2, TBA, FPG, and CREA were the top important factors (Fig. 2A). Additionally, we employed UMAP [20] to analyze the collection feature. By randomly extracting 1000 clinical and biochemical samples from our dataset, we observed that the features we collected were effective in predicting the risk of GDM (Fig. 2B). Furthermore, we calculated the Pearson correlation coefficients between all variables over the vectors of all samples. Our results demonstrated that A1MG, CysC, FPG, and BMG had a positive correlation with GDM while TP, ALB, TBIL, DBIL, UREA, and CO2 had a negative correlation with GDM (Fig. 2C). By integrating these findings, we have identified critical features that could be used for the early detection of GDM in pregnant women.

### Performance evaluation of the machine learning models

**Fig. 3:**
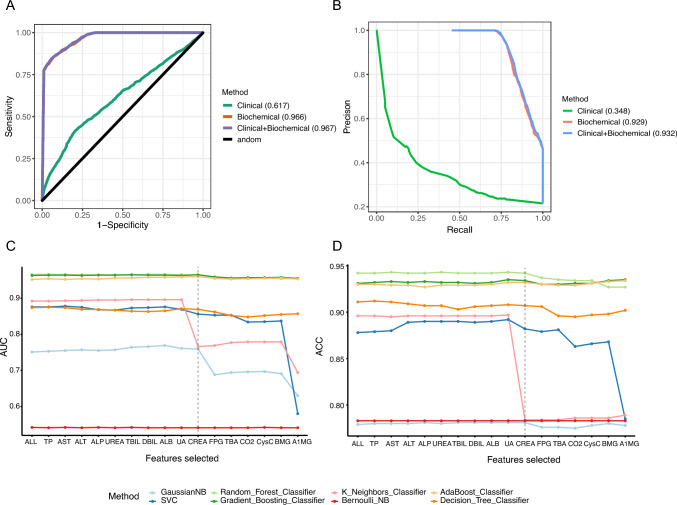
Performance evaluation of the GDMPredictor. **A** Area under the receiver-operating characteristic curve (AUC) for clinical, biochemical, and combined clinical and biochemical features. **B** Area under the precision–recall curve (auPRC) for clinical, biochemical, and combined clinical and biochemical features. **C** AUC for feature selection. **D** Accuracy (ACC) for feature selection

The GDMPredictor predicts the risk of GDM using the Random Forest (RF) method. To train the model, clinical features, biochemical features, and a combination of both were used. The AUC values obtained for models trained on only clinical features, only biochemical features, and clinical + biochemical features were 0.617, 0.966, and 0.967, respectively (Fig. 3A). Precision and recall curves indicated that models trained on biochemical and clinical + biochemical features performed better than the one trained on only clinical features (clinical auPRC = 0.348, biochemical auPRC = 0.926, and clinical + biochemical auPRC = 0.932) (Fig. 3B). Thus, the results revealed that models trained on clinical + biochemical features outperformed those trained on either biochemical or clinical features alone.

As the developed GDM predictor required user input of clinical and biochemical features, the model was designed to eliminate unimportant features (Fig. 3C and D). Biochemical features were removed in order of their importance and the performance of the model was tested using eight machine learning classification methods. Results showed that Random Forest, Gradient Boosting, and Adaboost outperformed the other five classification methods. Finally, we chose the best-performing RF model for our prediction model. During the model performance test, it was observed that the AUC and ACC performance results significantly decreased after the removal of creatinine CREA among the unnecessary biochemical features. Consequently, the following seven biochemical features: A1MG, BMG, CysC, CO2, TBA, FPG, and CREA were retained as the inputs for the prediction model.

### Important biochemical features and treatment recommendation

**Fig. 4:**
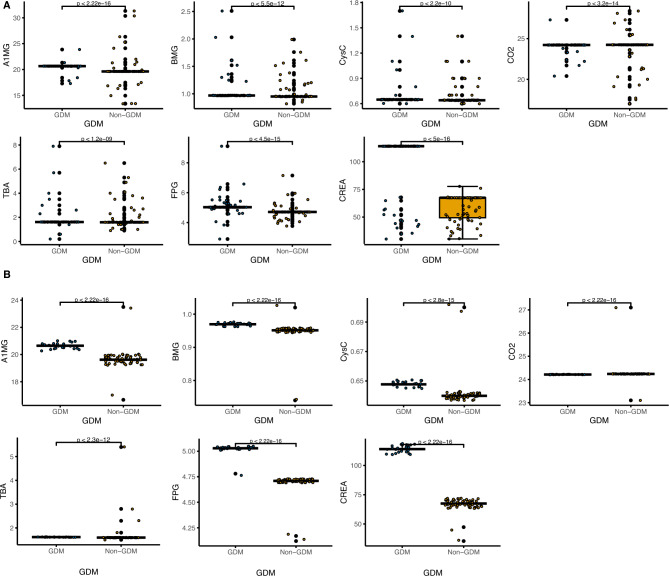
Top seven significant biochemical features among pregnant women during their first and twenty-fourth weeks of pregnancy. **A** Comparison of GDM and non-GDM biochemical values for A1MG, BMG, CysC, CO2, TBA, FPG, and CREA in the first week of pregnancy. **B** Comparison of GDM and non-GDM biochemical values for A1MG, BMG, CysC, CO2, TBA, FPG, and CREA in the 24th week of pregnancy. The X-axis represents the GDM group and the Non-GDM group, and the Y-axis represents the values of various biochemical indicators

**Fig. 5:**
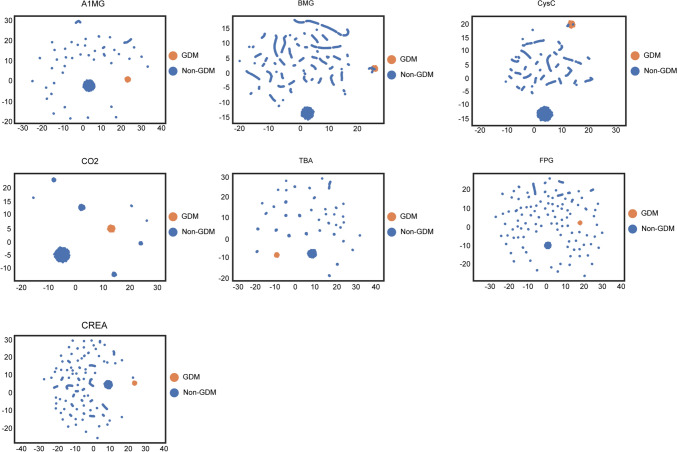
UMAP visualization of the top seven significant biochemical features. UMAP visualization highlighting the intricate relationships among the A1MG, BMG, CysC, CO2, TBA, GPG, and CREA significant biochemical features from our study

**Fig. 6:**
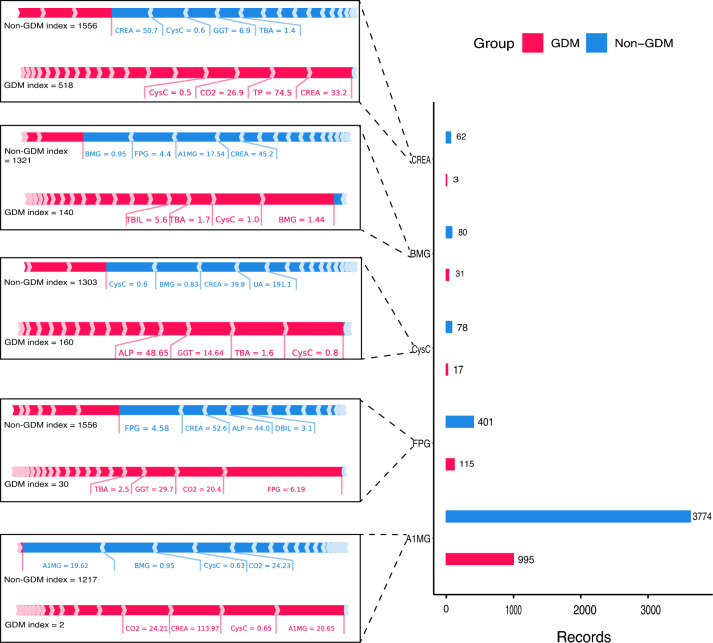
SHAP value interpretation of the top five significant biochemical features. The right side of the figure shows the statistics of the importance of different biochemical eigenvalues for the classification of GDM and Non-GDM, and the left side uses SHAP to show specific instances where the features affect the classification

In our study, we conducted a comparison of the top seven important biochemical features among pregnant women during their first and twenty-fourth weeks of pregnancy (Fig. 4). Our findings indicated that there were significant differences in A1MG, BMG, CysC, FPG, and CREA values at 24 weeks of gestation compared to those in early pregnancy. These differences were observed to be significantly higher. Additionally, we noted a trend of a significant increase in the biochemical values of GDM patients as compared to non-GDM ones. In contrast, CO2 values of both GDM and non-GDM subjects displayed a decreasing trend at 24 weeks of gestation, when compared to the first trimester of pregnancy. This observation is graphically presented in Fig. 4B Furthermore, we examined the interpretation of these seven important biochemical feature values separately by UMAP, which revealed that all these specific folds were able to distinguish significantly between GDM and non-GDM at various latitudes (Fig. 5). This finding supports the use of these biochemical features as potential predictors of GDM. Additionally, we scanned all the records statistically and found that the top five features of factors influencing GDM and non-GDM were A1MG, FPG, CysC, BGM, and CREA (Fig. 6). The results show that the main influence on GDM classification comes from the biochemical feature values. We also provided corresponding treatment recommendations for these GDM caused by biochemical values.

## Discussion

The GDMPredictor web server has the potential to greatly impact the prediction and treatment of GDM. By utilizing machine learning algorithms, the web server can accurately predict the risk of developing GDM based on a range of clinical and biochemical factors. This information can be invaluable in helping healthcare providers identify high-risk individuals early in their pregnancy, enabling them to provide appropriate interventions and management strategies to improve maternal and fetal outcomes. Furthermore, the GDMPredictor web server could also help healthcare providers in resource-limited settings where access to specialized testing and expertise may be limited. In these settings, the web server can serve as a cost-effective and efficient tool for predicting GDM risk and providing treatment recommendations. It is important to note that the GDMPredictor web server is not intended to replace clinical judgment or individualized medical care. Rather, it should be used as a supplementary tool to aid in decision-making and improve patient outcomes. Future studies should be conducted to evaluate the effectiveness and accuracy of the GDMPredictor web server in clinical practice. Medical decision support systems (MDSS) have been developed to assist clinicians in making more accurate diagnoses and treatment decisions [22]. The development of GDMPredictor, a web server for predicting the risk of GDM based on machine learning, is an example of such an MDSS. Compared to other MDSS, GDMPredictor has several unique features. The GDMPredictor uses a comprehensive set of clinical variables, including biochemical indicators and clinical history characteristics, to predict the risk of GDM. This is in contrast to some other MDSS that rely on a limited number of variables such as only using clinical information [23–25] or biochemical indicators [25–27], which may result in less accurate predictions. Furthermore, GDMPredictor provides treatment suggestions based on the predicted risk of GDM, which may improve the management of GDM. This is a unique feature not available in many other MDSS, which only provide predictions without treatment recommendations. Additionally, GDMPredictor is available as a web server, which can be accessed by healthcare providers and patients from anywhere with an Internet connection. This is a significant advantage over some other MDSS that require specialized hardware or software to be installed locally, limiting their accessibility and scalability.

Despite the promising results of the GDMPredictor web server, some limitations should be taken into account. First, the data used for training the machine learning model were collected from a single center, which may limit the generalizability of the model to other populations with different demographic and clinical characteristics. Second, some variables, such as family history, were not included in the model due to the lack of data, which may affect the accuracy of the prediction. We did not add racial features in the prediction, because we used data from a single center of the Asian population for model training Third, although the web server provides treatment suggestions based on the predicted risk, the final decision on the treatment plan should be made by a healthcare professional, taking into account the individual patient’s medical history and other factors. Finally, there is the lack of external validation of the predictive models used in GDMPredictor. External validation is crucial for assessing the generalizability and reliability of the predictive models. Future studies should include external validation of the GDMPredictor models using data from different populations.

To address these limitations, future studies should aim to collect more diverse and comprehensive data to improve the accuracy and generalizability of the machine learning model. Such as, placental proteomic as a factor to improve the accuracy [9]. The development of a mobile application version of the web server may increase its accessibility and usability for both patients and healthcare providers. Overall, the GDMPredictor web server has the potential to improve GDM prediction and treatment outcomes, as well as to advance the field of machine learning-based medical decision support systems. However, future work is needed to address the limitations of the current version of the web server and to explore its potential for wider application and impact.

### Supplementary Information

Below is the link to the electronic supplementary material.

Supplementary file1 (XLSX 818 KB) Supplementary Table 1. The trained data of clinical and biochemical features.

## Data Availability

The GDMPredictor can be accessed from http://www.bioinfogenetics.info/GDM/.

## Abbreviations

GDM: Gestational diabetes mellitus
OGTT: Oral glucose tolerance test
IADDPSG: International Association of Diabetes and Pregnancy Study Groups
SVM: Support vector machine
AUC: Area under the curve
ROC: Receiver-operating characteristic curve
TPR: True-positive rate
FPR: False-positive rate
RF: Random forest
AUC: Area under the ROC curve
TP: True positive
FP: False positives
FN: False negatives
TN: True negatives
AP: Adverse pregnancy
ICP: Intrahepatic cholestasis of pregnancy
TD: Thyroid diseases
ALT: Alanine aminotransferase
AST: Aspartate aminotransferase
GGT: Gamma-glutamyl transferase
ALP: Alkaline phosphatase
TP: Total protein
ALB: Albumin
TBIL: Total bilirubin
DBIL: Direct bilirubin
TBA: Total bile acid
UREA: Blood urea nitrogen
CREA: Creatinine
UA: Uric acid
BMG: Blood glucose
A1MG: Alpha-1 microglobulin
CysC: Cystatin C
CO2: Carbon dioxide
SHAP: Shapley values
UMAP: Uniform Manifold Approximation and Projection for Dimension Reduction
auPRC: Area under the precision–recall curve
MDSS: Medical decision support systems
